# Synergistically Enhancing the Therapeutic Effect on Cancer, via Asymmetric Bioinspired Materials

**DOI:** 10.3390/molecules27238543

**Published:** 2022-12-04

**Authors:** Yasamin Ghahramani, Marzieh Mokhberi, Seyyed Mojtaba Mousavi, Seyyed Alireza Hashemi, Fatemeh Fallahi Nezhad, Wei-Hung Chiang, Ahmad Gholami, Chin Wei Lai

**Affiliations:** 1 Department of Endodontics, Dental School, Shiraz University of Medical Sciences, Shiraz 7195615787, Iran; 2 Dentist, Dental School, Shiraz University of Medical Sciences, Shiraz 7195615787, Iran; 3 Department of Chemical Engineering, National Taiwan University of Science and Technology, Taipei 106335, Taiwan; 4 Nanomaterials and Polymer Nanocomposites Laboratory, School of Engineering, University of British Columbia, Kelowna, BC V1V 1V7, Canada; 5 Oral and Dental Disease Research Center, School of Dentistry, Shiraz University of Medical Sciences, Shiraz 7195615787, Iran; 6 Biotechnology Research Center, Shiraz University of Medical Sciences, Shiraz 7146864685, Iran; 7 Nanotechnology and Catalysis Research Centre (NANOCAT), Institute for Advanced Studies (IAS), University of Malaya (UM), Kuala Lumpur 50603, Malaysia

**Keywords:** cancer, bioinspired nanomedicine, biomimetic materials, targeted drug delivery, asymmetry

## Abstract

The undesirable side effects of conventional chemotherapy are one of the major problems associated with cancer treatment. Recently, with the development of novel nanomaterials, tumor-targeted therapies have been invented in order to achieve more specific cancer treatment with reduced unfavorable side effects of chemotherapic agents on human cells. However, the clinical application of nanomedicines has some shortages, such as the reduced ability to cross biological barriers and undesirable side effects in normal cells. In this order, bioinspired materials are developed to minimize the related side effects due to their excellent biocompatibility and higher accumulation therapies. As bioinspired and biomimetic materials are mainly composed of a nanometric functional agent and a biologic component, they can possess both the physicochemical properties of nanomaterials and the advantages of biologic agents, such as prolonged circulation time, enhanced biocompatibility, immune modulation, and specific targeting for cancerous cells. Among the nanomaterials, asymmetric nanomaterials have gained attention as they provide a larger surface area with more active functional sites compared to symmetric nanomaterials. Additionally, the asymmetric nanomaterials are able to function as two or more distinct components due to their asymmetric structure. The mentioned properties result in unique physiochemical properties of asymmetric nanomaterials, which makes them desirable materials for anti-cancer drug delivery systems or cancer bio-imaging systems. In this review, we discuss the use of bioinspired and biomimetic materials in the treatment of cancer, with a special focus on asymmetric nanoparticle anti-cancer agents.

## 1. Introduction

**Figure 1:**
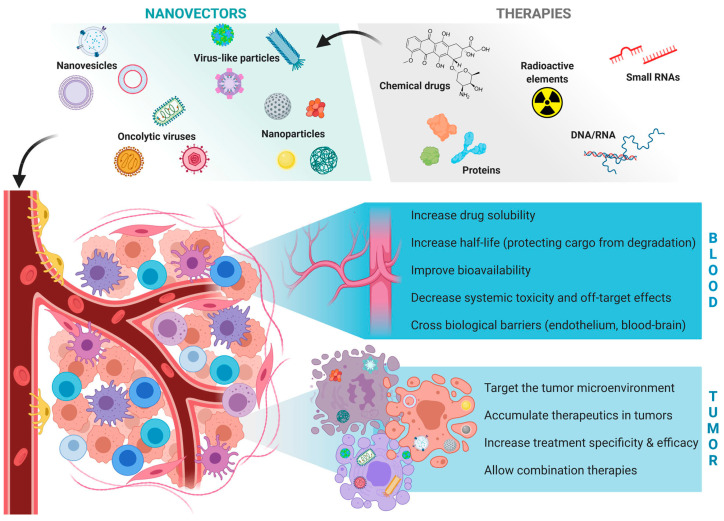
The advantages of applying nanocarriers for delivering cancer therapies [11].

Cancer is a term that encompasses a variety of diseases caused by the uncontrolled growth of malignant cells that can invade and spread to other parts of the body. The World Health Organization estimates that there will be 13.1 million cancer-related deaths by 2030, with more than 10 million new cases reported each year [1,2]. For both genders, lung cancer is the most commonly diagnosed cancer and the leading cause of cancer deaths. Breast cancer, prostate cancer, and colorectal cancer are other cancers commonly diagnosed in women [3]. The survival rate of cancer patients has been ameliorated with the development of an understanding of cancer physiology and its treatment modalities, such as chemotherapy, radiotherapy, and immunotherapy. However, due to the lack of tumor specificity of these treatment methods, they can induce off-target side effects in healthy tissues as well as limited efficacy due to insufficient drug concentration at the tumor sites [4,5,6]. Moreover, in the case of resistance to single therapies, using combination therapies is essential, which can increase the risk of lethal side effects. Additionally, some cancer drugs, such as radioisotopes, toxins, nucleic acids, or hydrophobic drugs, cannot be administered systemically. Therefore, in order to overcome the mentioned limitations, targeted drug delivery using nanocarriers has been developed (Figure 1). Specific targeting augments drug solubility and bioavailability, increases drugs’ stability, and improves drug targeting and its concentration in the tumor site [7,8,9,10].

Over the past few decades, different types of nanomaterials have been well developed for cancer therapies. The employed nanomaterials can be produced in symmetric or asymmetric structures. The nanomaterials are commonly synthesized in symmetric forms, such as nanospheres, nanowires, nanoflowers, or nanosheets. Additionally, after the discovery of Janus by Gennes et al. in 1991 [12], the asymmetric structures have gradually attracted increasing attention; thus, various asymmetric structures have been discovered in the last two decades. Nowadays, asymmetric structures contain various shapes with different surface properties [13,14,15]. Janus particles, bowl-shaped [16], snowman-shaped [17], disk-shaped [18], and raspberry-shaped structures [19], are examples of asymmetric nanomaterials. An asymmetric particle is a particle with an asymmetric center, similar to an asymmetric molecule. Different surface properties (e.g., charge, polarity, and chemical functionality) and particle shapes (e.g., dumbbells, snowmen, and Janus particles) can be responsible for the particle’s asymmetry [20]. The application of nanomaterials in cancer therapy has various advantages due to their physicochemical properties, such as nanometric dimensions, large surface area-to-volume ratio, tunable surface characteristics, the ability to encapsulate various molecules, and controlled drug release [8,21,22,23]. Additionally, nanomedicine increases the therapeutic molecules’ stability, bioavailability, and tumor accumulation, resulting in a prolonged half-life in circulation compared to conventional chemotherapic drugs [24,25,26,27]. These properties make nanomedicines an excellent choice in tumor-targeted treatments. Despite the mentioned advantages and applications of nanomaterials in cancer therapy, some shortages are associated with these materials. In preclinical studies, the application of drug-loaded nanocarriers results in high drug concentrations in tumor sites with maximum therapeutic efficacy, while in clinical studies, the targeted synthetic nanoparticles (NPs) cannot work due to impenetrable biological barriers [8,28]. Therefore, scientists have been developing nanomedicine mimicking biological features with the inspiration of natural structures to overcome biological barriers. Additionally, synthetic nanomaterials can be modified with some biomimetic features. These bioinspired nanomedicines possess desirable properties such as excellent biocompatibility, proper biodegradation, and the ability to deliver high drug-loading content to target cells [29,30,31]. Therefore, bioinspired materials are a new solution to overcome biological barriers and the disadvantages of current drug delivery systems (DDSs). This review will discuss the application of asymmetric nanomaterials in cancer treatment.

## 2. Asymmetric Nanomaterials

As mentioned before, both symmetric and asymmetric structure nanomaterials are employed for cancer treatment. The symmetric structures (i.e., nanospheres, nanowires, nanoflowers, nanosheets, core–shell structured composites) are now commonly used in biomedical applications. Additionally, after the discovery of Janus by Gennes et al. in 1991 [12], the asymmetric structures have gradually attracted increasing attention; thus, various asymmetric structures have been discovered in the last two decades. Nowadays, asymmetric structures contain various shapes with different surface properties [13,32,33].

While the surface free energy effect limits symmetric structures, various unique advantages are related to asymmetric structures (Table 1). One of the most practical advantages of asymmetric structures is that they can possess multiple functions due to their different surface physiochemical properties or components. This results in the ability of these nanomaterials to simultaneously contain distinct properties (e.g., hydrophilicity and hydrophobicity), which makes the asymmetric structures ideal candidates for “nano-intelligent systems” with desirable applications in biomedicine, electrochemistry, and interfacial stabilizers [13,34,35]. Furthermore, another advantage of asymmetric structures is a stronger synergistic effect. The distinct parts of asymmetric structures can perform independently or can even cooperate, resulting in enhanced efficiency. Additionally, because of their asymmetric shape, they have a larger effective surface area with an increased number of active sites, resulting in more preferred properties of these materials. Therefore, asymmetric materials have been widely developed in recent years due to their desirable properties [13]. Because of the interaction between asymmetric shapes and directional interactions, asymmetric particles with anisotropic features can yield more complex compositions than symmetric particles. The device usually has a symmetric geometry, and there is a limited amount of space available to load multiple drug types into the device. If two drugs are loaded simultaneously into a single storage space, the release of each drug cannot be independently controlled. In addition, the loaded multidrugs may interact adversely with each other, especially if the drugs have different chemical properties (e.g., hydrophilicity and hydrophobicity, acidity and basicity, etc.). Therefore, the development of multicompartment carriers with independent storage sites to accommodate multiple drugs is urgently needed. Dual surface structures of an asymmetric nanostructure are anisotropic in composition, shape, and surface chemistry, which makes them ideal for binding dual guests to different domains of asymmetric particles. In addition, the functionally different surfaces of asymmetric particles can also be used for selective conjugation with specific triggers, allowing the release of the double guests to be individually controlled [36,37,38].

### 2.1. Janus Nanoparticles

**Figure 2:**
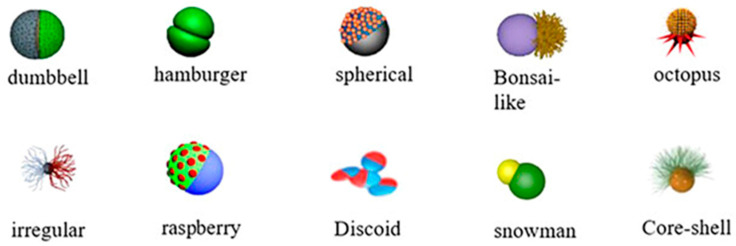
The schematic illustration of some Janus nanoparticles [44].

Janus was the first asymmetric particle introduced by Pierre-Gilles Gennes in 1991 as a particle with two sides with opposite polarity [12]. With the development of nanotechnology, Janus nanoparticles (JNPs) are fabricated as NPs with two or more sides that have distinct chemical or physical properties. Generally, JNPs are divided into three groups due to their composition. JNPs can be organic (e.g., polymeric), inorganic (e.g., gold, silver, silica), or organic–inorganic [39,40,41,42,43]. Bowl-shaped [16], snowman-shaped [17], disk-shaped [18], raspberry-shaped [19], hamburger, spherical, bonsai-like, octopus, core-sell, and irregular structures [44] are the various shapes of JNPs (Figure 2).

Janus nanoparticles have attracted attention in cancer treatment due to their heterogeneous structure, as they can participate in two distinct functions. JNPs can corporate various materials in order to achieve specific properties. As an example, with the incorporation of JNPs with imaging materials such as MnO_2_, Fe_3_O_4_, gold and silver NPs, fluorescent dyes, or quantum dots, various imaging modalities are designed for tumor cell screening [45,46]. Several interesting properties can be obtained in nanoparticles with asymmetric heterostructural compounds at the micro/nanoscale that are not possible in homogeneous or symmetric nanostructures. For example, the Fe_3_O_4_-Au JNPs exhibited magnetic properties on one side, while the Au NPs showed localized surface plasmon resonance on the other side. The Janus structure allowed Fe_3_O_4_ to be combined with Au, resulting in a different surface polarity or internal chemistry than the corresponding single components [47,48,49].

Moreover, incorporating JNPs with therapeutic agents can be employed as nanocarriers in various therapeutic modalities for cancer treatment, such as chemotherapy, phototherapy, radiotherapy, and gene therapy. In the drug delivery field, JNPs can be loaded with various distinct drugs with an independent release of multiple drugs [50], as well as acting as nanomotors for active drug delivery or physical tumor destruction [51,52,53]. Therefore, the Janus nanoparticles have gained attention as the new nanoparticles in the cancer diagnosis and treatment era (Table 2).

#### 2.1.1. Polymeric Janus Nanoparticles

**Figure 3:**
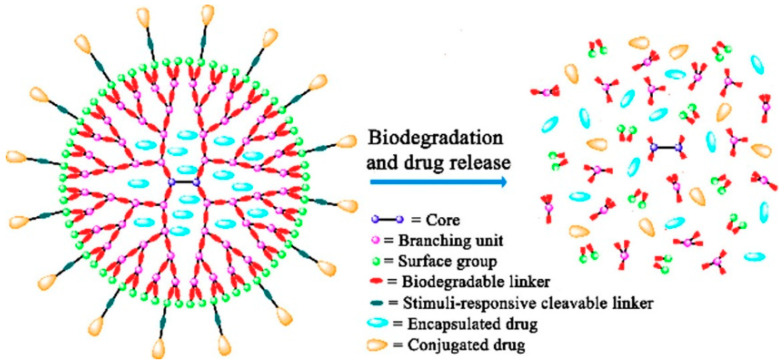
Schematic illustration of the biodegradation of biodegradable dendrimers [59].

In recent years, polymeric JNPs have been introduced as a drug delivery agent in cancer treatment. Janus dendrimers and spherical polymeric nanoparticles are the most common JNPs in drug delivery systems (DDS). Dendrimers are known as branched macromolecules with a core that is surrounded by repetitive branching units, known as “Dendron”. Janus dendrimers are a group of favorable drug carriers due to their properties, such as hydrophilicity, drug encapsulation ability, and the ability to conjugate with drugs with their abundant functional groups [54,55,56]. Dendrimers are categorized as asymmetric nanomaterials as they can have two or more distinct dendrons; thus, unlike symmetrical NPs, they can have more than one functional ability. As an example, Acton et al. fabricated polyethylene glycol (PEG)-based Janus dendrimers which can be loaded with two distinct hydrophobic and hydrophilic drugs. Additionally, the mentioned dendrimers are able to release the drugs at different speeds due to the different linkages between the dendrons and the drugs (Figure 3) [57,58,59].

The polymeric Janus materials are also applicable in cancer treatment. As the studies showed with the elimination of drug carriers in blood circulation by macrophages and neutrophils, polymeric Janus particles were introduced to overcome this problem. For instance, Sanchez et al. designed two-sided structures of polyethylene glycol (PEG) chains and Janus particles coupled with IgG, which reduced interference between the distinct functions of the two sides of JNPs by a spatial decoupling design of NPs, while the PEGs decrease the effect of macrophage evasion. Additionally, the mentioned study demonstrated that articles coated with only half PEG could escape from the immune system more effectively than particles coated with full PEG [60,61,62]. According to Xie and colleagues, biocompatible polymeric Janus nanoparticles composed of the FDA-approved polymer poly-(lactate-co-glycolic acid) (PLGA) can be prepared in one step using a fluidic nanoprecipitation system (FNPS). It was the first report of polymeric Janus nanoparticles capable of carrying a hydrophobic drug (paclitaxel) and a hydrophilic drug (doxorubicin hydrochloride) in a single particle [63,64].

#### 2.1.2. Inorganic Janus Nanoparticles

**Figure 4:**
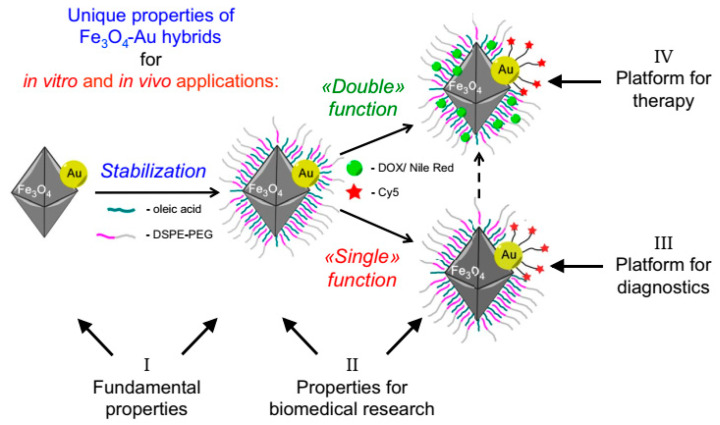
Schematic illustration of Au-Fe_3_O_4_ multifunctional Janus nanoparticles as a cancer diagnostic and therapeutic agent [74].

Inorganic materials such as gold, silver, silica, MnO_2,_ or Fe_3_O_4_ can be utilized in the fabrications of Janus nanoparticles for cancer diagnosis and treatment (Figure 4) due to their physiochemical properties such as magnetism, surface plasmonic resonance (SPR), photo-thermal conversion ability, and easy functionalization [65]. Therefore, the inorganic-based JNPs are mainly employed as cancer imaging agents in magnetic resonance imaging (MRI), magnetic particle imaging, computerized tomography (CT) scans, photoacoustic imaging processing (PAI), surface-enhanced Raman scattering (SERS), etc. [66]. In a study, Reguera et al. fabricated a star-shaped JNP that forms the initial Fe_3_O_4_–Au dumbbell JNPs. The fabricated nanostar can be utilized in MRI, CT, SERS, or PAI cancer imaging systems [67,68,69,70].

In addition to imaging systems, inorganic JNPs are employed in various cancer treatment modalities. In a study, the surface of silica-based JNPs was functionalized with alkyne-tagged anti-CD28 and biotinylated anti-CD3 antibodies, fabricating bifunctional Janus nanoparticles. As a result, the fabricated JNPs can be utilized as T-cell stimulation NPs in immunotherapies against tumors, containing both anti-CD3 and anti-CD28 stimulatory ligands in two distinct sides of JNPs [71]. In another study, gold-mesoporous silica JNPs were designed and then modified with folic acid; thus, the fabricated JNPs can be employed as drug nanocarriers. In the mentioned study, the gold-mesoporous silica JNPs were loaded with doxorubicin (DOX) (an anti-cancer chemotherapy drug), which can be released from the mesoporous agents in a pH-sensitive manner, targeting and entering hepatocellular carcinoma tumor cells. In addition to chemotherapy, the gold-mesoporous silica JNPs can act both as radiotherapy and CT-scan agents due to the physicochemical properties of inorganic gold and silica molecules, as mentioned before [72,73].

Despite the challenges facing molecular organic-inorganic hybrid mesoporous organosilica nanoparticles (MONs), the precise control of morphology, nanostructure, composition, and particle size remains a challenge. It has been shown that hollow MOS nanoparticles (HMONs) with uniform spherical morphology can be prepared using a hard template. An efficient growth strategy governed by bridged organic groups was proposed for the facile synthesis of highly dispersed and uniform MONs with different Janus morphologies, nanostructures, organic-inorganic hybrid compositions, and particle sizes. As long as the bridged organic groups and the concentration of bis-silylated organosilica precursors are varied, the properties of MONs can be easily controlled. In addition to their excellent performances as stimuli-responsive drug carriers, adsorbents for bilirubin removal, and contrast agents for ultrasound imaging, Janus MONs have hollow structures [1,75].

#### 2.1.3. Polymeric-Inorganic Janus Nanoparticles

**Figure 5:**
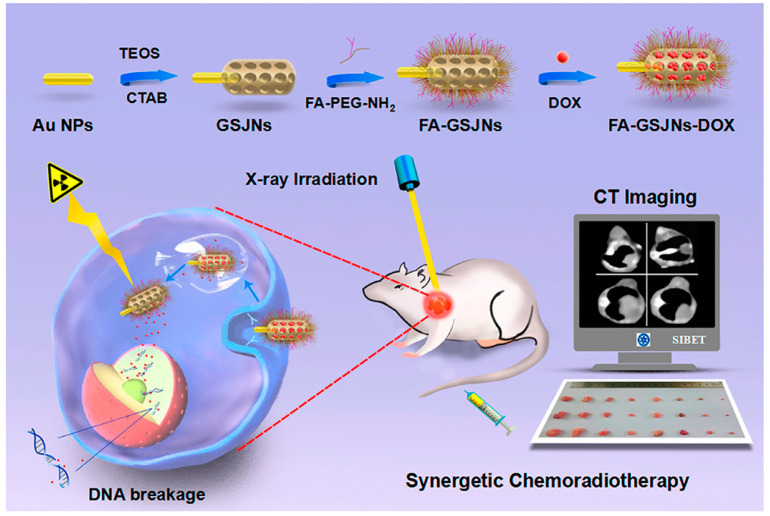
A multifunctional gold-mesoporous silica Janus nanoparticle designed for a bio-imaging system [72].

In addition to polymeric and inorganic JNPs, a new category of Janus nanoparticles was also designed by combining these two mentioned JNPs, named polymeric–inorganic Janus nanoparticles. These JNPs are widely employed for biomedical applications, including cancer diagnosis and treatment. In a study, a polystyrene/Fe_3_O_4_@SiO_2_ Janus nanocomposite was designed by Wang et al. for administering targeted drug delivery. The mentioned JNP was further loaded with folic acid on the polystyrene side and doxorubicin on the silica side [76,77,78]. Additionally, with the application of Fe_3_O_4_ nanoparticles, and due to their unique properties, simultaneous targeting, drug delivery, and imaging can be performed. In another study, gold-mesoporous silica Janus nanoparticles were employed in a bio-imaging system (Figure 5). In these multifunctional JNPs, the nanoparticles were designed to target hepatocellular Carcinoma due to the folic acid molecules in their structure. In other words, the folic acid modification of these JNPs enables them to act as targeted computed tomography (CT) and imaging agents for diagnosing hepatocellular Carcinoma [72,79].

**Table 2:** The examples of employed JNPs in the studies related to cancer diagnosis and treatments.

Type	Composition	JNPs Morphology	Application	Reference
Organic	Four PEG-based dendrons	Dendrimers	Chemotherapy	[57]
	PLGA nanoparticles-DOX-PTX	Sphere	Chemotherapy	[63]
Inorganic	Silica-antibodies	Sphere	Immunotherapy	[71]
	Fe_3_O_4_-Au	Octahedron-sphere/Star	CT, MRI, Chemotherapy, PIA, SERS	[67,74]
	Au-silica	Sphere	PIA	[80]
	FA-Au-mesoporous silica-DOX	Spindle	CT, Chemotherapy, Radiotherapy	[72]
	GNRs@mSiO_2_–DOX	Lollipop	Chemotherapy	[81]
	FA-Au/Fe_3_O_4_@C	Dumbbell	MRI, CT, Chemotherapy	[82]
	DOX-CMR-MS/Au-6MP	Dumbbell	Chemotherapy, SERS	[83]
	Fe_3_O_4_-MSNs-P@GCV@pTK	Rod	MRI, Magnetic hyperthermia, Gene therapy	[84]
	Fe_3_O_4_-SiO_2_	Bullet	Magnet field-enhanced chemotherapy	[85]
	Ag-MSN-DOX	Bullet	Chemotherapy	[86]
polymeric-inorganic	Fe_3_O_4_-PS16-PAA10	Sphere	Chemotherapy	[87]
	FA-Polystyrene/Fe_3_O_4_@SiO_2_-DOX	Sphere	Chemotherapy	[87]
	Au-polydivinylbenzene-curcumin	Sphere	Chemotherapy	[88]
	FA-Au-PAA/mCaP	Dumbbell	CT, Chemotherapy	[89]

### 2.2. Asymmetric Mesoporous Materials

**Figure 6:**
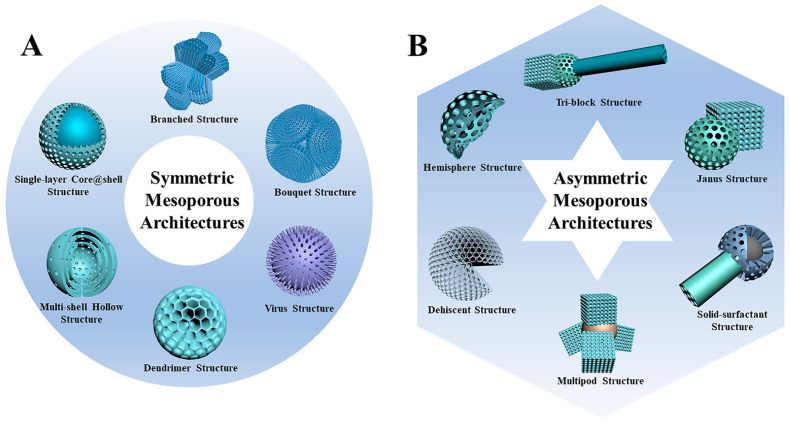
Schematic illustrations of (**A**) symmetric and (**B**) asymmetric mesoporous nanoparticles with various architectures [95].

Mesoporous materials are materials with 2–50 nm diameter pores, which have been widely developed over the past decades [80,90,91]. The first mesoporous materials were bulk, but with their increased application in various fields, nano-sized mesoporous materials were fabricated, known as mesoporous nanoparticles [92,93,94]. Nowadays, mesoporous NPs are widely employed in various domains, including biomedicine and drug delivery, due to their large surface area, high pore volume, and tunable pore size, with various structures and components. The mesoporous NPs are fabricated in unique symmetric and asymmetric architectures, as shown in Figure 6.

Despite the symmetric NPs, asymmetric mesoporous NPs are designed for multiple-component drug-loading systems or ligand attachment due to having more than one distinct structure. Moreover, each mesoporous component can contain different pore sizes, particle sizes, electric charges, hydrophilicity, etc.; therefore, each component is able to interact independently with the surrounding environment. This characteristic makes the asymmetric mesoporous nanoparticles a favorable material for biomedical applications and drug delivery systems. In a study, Li et al. fabricated multifunctional asymmetric UCNP@SiO_2_@mSiO_2_&PMO (UCNP = upconversion nanoparticle, PMO = periodic mesoporous organosilica) nanoparticles. These NPs contain a hydrophilic side (mSiO_2_) and a hydrophobic side (PMO); therefore, the NPs can be loaded with hydrophilic doxorubicin drugs on the mSiO_2_ side, and with the hydrophobic paclitaxel molecules on the PMO side. These two drugs can be released independently due to heat production with NIR light [36]. Mesoporous silica nanoparticles with anisotropic geometry and dual compartments are highly desirable for loading and unloading dual drugs in segregated storage environments. An anisotropic epitaxial growth strategy has successfully developed an asymmetric lollipop-shaped mesoporous silica nanoparticle Fe_3_O_4_@SiO_2_&EPMO (EPMO = ethane-bridged periodic mesoporous organosilica). An asymmetric nanoparticle exhibits a uniform lollipop shape with a 200 nm diameter spherical core of iron3O_4_@SiO_2_ and a 90 nm-long EPMO nanorod with a specific surface area of 650.3 square meters g-1. According to in vitro studies, the asymmetric nanoparticles possess a unique dual independent (hydrophilic/hydrophobic) space design with high loading capacity and are significantly more effective than pure drugs at killing cancer cells. In addition, the dual drug-loaded nanoparticles (curcumin plus gentamicin sulfate) exhibited excellent antibacterial and anticancer properties [96,97,98].

The asymmetric nanoparticles of mesoporous silica have a head-to-tail structure and are potent immunoadjuvants capable of delivering peptide antigens to mice and eliciting greater antibody immune responses than their symmetric counterparts. According to Abbaraju et al., asymmetric mesoporous silica nanoparticles with a head-to-tail morphology (HTMSN) were more effective than symmetric mesoporous silica nanoparticles (MSN) when using a peptide antigen as an adjuvant. Recently, it has been reported that HTMSNs have been synthesized and have the potential to be used as an adjuvant in vitro. To demonstrate the efficacy of HTMSNs as vaccine adjuvants, researchers used J8 (molecular weight 3283) as a model antigen that can be efficiently loaded in both MSNs and HTMSNs. The J8 peptide, developed from the bacterial surface M protein, generates antibody-specific immunity and protection against Streptococcus pyrogenes (SP), an infection known for its numerous clinical manifestations, including toxic shock syndrome, rheumatic fever, and rheumatic heart disease (RHD). In studies using MSN and HTMSN as both the adjuvant and carrier, HTMSN showed enhanced uptake into antigen-presenting cells (APCs) and the increased activation of costimulatory molecules (e.g., CD40, CD86) on the surface of matured APCs compared with MSN. Another study in mice found that HTMSN loaded with J8 elicited a stronger response to J8-specific antibodies than MSN [99,100]. In another study, asymmetric single-hole mesoporous silica nanocages were designed. These nanoparticles are hollow spheres of 100–240 nm diameter, with mesoporous shells on the surface area. These NPs can be loaded with drugs of various sizes, due to their central hollow area (∼25 nm) and the mesopores in silica molecules with 2–10 nm diameter. In this study, the mesoporous silica NPs were loaded with both bovine serum albumin and doxorubicin, with two different particle sizes. These molecules can also be released separately, controlled by heat and NIR light [101,102,103].

## 3. Core–Shell Nanoparticles

Core–shell nanoparticles are a type of asymmetric nanoparticles with distinct components in an inner core and outer shell, which makes this nanoparticle able to attach to both nanometric and micrometric scales. More importantly, their particular structure results in a controlled releasing action while protecting the drug molecules. Due to their application and purpose, core–shell NPs can be fabricated in different core shapes, shell structures and thicknesses, and surface properties. The mentioned characteristics can determine the loading capacities and releasing kinetics of drugs. The core–shell NPs usually vary between 10–200 nm, as the smaller particles will be inactivated by immune cells, and the larger ones will be recognized as foreign body particles, causing inflammation [104,105].

Various core–shell nanoparticles have been employed successfully as anti-cancer drug delivery agents. In the studies, magnetic NPs, gold NPs, Fe_3_O_4,_ and other conducting NPs are mainly used as core materials. As an example, Ayyanaar et al. employed Fe_3_O_4_ nanoparticles as the core material, coated in poly(lactic-co-glycolic acid) (PLGA) mesoporous molecules, fabricating PLGA-Fe_3_O_4_ nanoparticles. These nanoparticles were loaded with curcumin which is an anti-cancer agent [106].

## 4. The Applications of Asymmetric Nanomaterials in Cancer Treatment

As mentioned, asymmetric structures are employed for various applications due to their desirable properties, such as a more significant surface area, increased active sites, and tunable structures and compositions. Among the nanomaterials, carbon-based and silica-based asymmetric nanomaterials have been widely developed recently due to their advantages, such as favorable biocompatibility, facile application, and structural adjustability [29,107]. Additionally, asymmetric nanomaterials have been designed for biomedical applications because of their ability to integrate different functional components, structures, and even properties. In this regard, this section will thoroughly discuss the application of asymmetric nanomaterials in cancer treatment.

### 4.1. Drug Delivery

Enhancing drug delivery systems (DDS) is one of the significant applications of asymmetric nanomaterials in cancer treatment. The tumor-targeted drug delivery of anti-cancer drugs has been developed in past decades to achieve a controlled drug-releasing system to increase the treatment efficacy and decrease the off-target effects. Nanomaterials are considered favorable drug carriers in cancer therapy. In addition, asymmetric nanomaterials are more efficient as their distinct components can function separately. For example, Shao et al. designed a bullet-like nanoparticle (NP) with a head of Fe_3_O_4_ and a body of mesoporous silica [85,108]. In this nanoparticle, the magnetic Fe_3_O_4_ enables an increased drug concentration and enhanced cellular uptake using magnetic field-guided tumor accumulation. Additionally, the mesoporous silica enables the enhanced loading of the drugs. Moreover, the mentioned NP can be loaded with various molecules. The Janus Fe_3_O_4_–mesoporous silica NP can be loaded with photosensitizers for combination therapies, while Fe_3_O_4_ is used for inducing magnetic hyperthermia combined with the photodynamic therapy of tumor cells to prevent metastasis [109]. In addition, the metallic part can be replaced by gold or silver NPs for reaching synergistic photo-thermal/chemotherapy applications due to the photo-thermal properties of noble NPs [110,111,112]. In another study, octopus-type gold nanostar-mesoporous silica asymmetric NPs were utilized. The silica segments of these NPs were conjugated with lactobionic acid (LA). This conjugation induces a higher drug capacity of the NPs, with affected drug release properties by pH and near-infrared (NIR), resulting in actively targeted chemo-photothermal therapy [113]. Moreover, asymmetric NPs are advantageous in cargo delivery. The conventional NPs do not have enough drug-loading capacity for large biomolecules like nucleic acids and proteins, while asymmetric NPs have higher cargo-loading efficiency. In a study, Qiao et al. designed bowl-shaped silica NPs with high loading capacity for plasmid DNA, applicable for DNA delivery systems [114,115].

In order to reach efficient drug delivery, the cellular uptake of these nanocarriers is also important. A suggested solution for increasing cellular uptake is using a rough surface head component. In the study by Li et al., they developed asymmetric nanotruck NPs with rough silica and a periodic mesoporous organosilica (PMO) rod, resulting in the enhanced intracellular uptake of the nanotruck compared to smooth-surfaced PMO [90]. Additionally, the upconverting nanoparticles (UCNPs) can be encapsulated with a rough silica head for a dual application of bio-imaging and NIR-triggered drug delivery [13]. This specific design suggests the potential of achieving various distinct applications for a single nanoparticle by developing new structural properties in asymmetric NPs.

**Figure 7:**
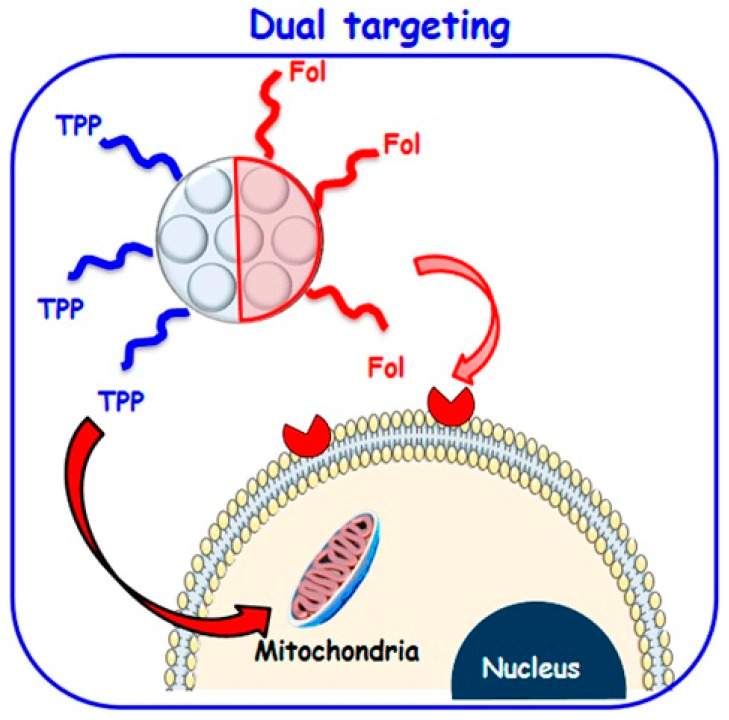
Dual targeting strategy to attack both tumor cell membrane and mitochondria by asymmetrically functionalized nanoparticles [118].

In addition, the unique properties of the asymmetric NPs make it possible to tailor them for a particular application like dual drug delivery. As an example, a dual-component asymmetric NP was developed by Zhao et al., using mesoporous silica and PMO for dual-drug delivery. Since mesoporous silica is hydrophilic and PMO is a hydrophobic component, this NP can be loaded with two hydrophilic and hydrophobic drugs without interfering [36,116]. Additionally, the outer surface of the asymmetric NPs can be modified with different functional groups. López et al. designed asymmetric Janus mesoporous silica particles with two different targeted ligands. One is a folic acid molecule for binding to the folate receptors of the cell membrane, and the other is triphenylphosphine for binding to the mitochondria membrane. This unique design results in a higher nanoparticle concentration inside the tumor cells due to folic acid ligands with the guided transportation of nanocarriers near the mitochondria with triphenylphosphine function [117]. This asymmetric designing strategy facilitates the targeted delivery of NPs from cell to organelle compared to conventional symmetric NPs. (Figure 7).

The asymmetric nanoparticles also apply in active drug delivery systems using nanomotors. The asymmetric structures with tunable properties enable the nanomotors to move in complex biological conditions to achieve efficient drug delivery. The studies suggest that the asymmetric silica-based motors can significantly penetrate cell membranes or tumor cells due to the autonomous motion of these nanomotors [119,120,121,122,123].

### 4.2. Tumor Imaging Systems

After the production of cancer cells in the human body, they can replicate rapidly, resulting in tumor formation. Additionally, these cancerous cells can transfer through the circulation system and metastasis in various organs. Therefore, developing tumor screening systems for the early detection of tumoral cells is essential. With the development of nanomaterials’ applications in biomedicine, they are widely employed for tumor detection by various screening modalities such as biosensors, multifunctional CT scans, or MRI systems.

Like other nanometric materials, asymmetric nanoparticles are also employed for tumor detection. Gold-integrated asymmetric NPs have great potential for application in radiosensitization and the computerized tomography (CT) imaging of tumors [72,124]. In a study, Wang et al. designed a carbon-based snowman-shaped asymmetric nanostructure with gold and Fe_3_O_4_, resulting in simultaneous CT/magnetic resonance imaging (MRI) and chemo-photothermal synergistic therapy in a single asymmetric nanostructure [82,125].

Detecting cancer biomarkers with biosensors is another method of tumor detection. Nanomaterials are commonly used in the structure of biosensors. As nanomaterials have high electrical conductivity, high affinity to biomolecules, and high surface area-to-weight ratios, they are known as desirable candidates in biosensor fabrication. The application of nanomaterials can optimize the signal transduction of biosensors due to the enhancement of sensitivity [126,127]. Therefore, it has resulted in the better selectivity of biomolecules, signal/noise ratio, and signal per effect due to multiple receptors, as well as decreasing the size of the electrochemical biosensors [128,129,130,131]. Designing the ideal electrode with high selectivity and sensitivity is a significant limitation of the biosensors, which conjugating NPs resolve to the biomarkers [132]. Additionally, choosing the proper functional group is essential for an ideal biosensor design. As mentioned, asymmetric NPs can bind with various molecules or modify with distinct functional groups due to their unique physical properties. Therefore, asymmetric nanomaterials can be utilized in the designing and production of cancer biosensors in order to achieve enhanced detection selectivity and specificity.

## 5. Bioinspired and Biomimetic Nanomaterials in Cancer Therapy

Despite the advantages of the nanoparticles in the targeted drug delivery systems, they contain low drug delivery efficacy due to improper biocompatibility and low permeability in biological barriers; thus, bioinspired and biomimetic nanomaterials are developed in recent years to overcome these shortages, resulting in higher bioavailability and an enhanced therapeutic effect. In this section, one of the most common bio-inspired materials in cancer therapy will be discussed. Among the vitamins, Vitamin B12 (VB12) is an ideal material for NP production. VB12 has receptor-mediated endocytosis in the human body and forms a complex with the intrinsic stomach matrix. For instance, Chalasani et al. designed a study in which the drug availability of covalent conjugation of VB12 to insulin-loaded dextran was compared with pure NPs. The results of this study indicated the higher pharmacological availability of VB12-conjugated drugs [133,134].

Vitamin B9, also known as folic acid (FA), is another vitamin with a significant affinity for folate receptors, resulting in an increased cellular uptake of tumor cells. In a study, a polystyrene/Fe_3_O_4_@SiO_2_ Janus nanocomposite was designed by Wang et al. for administering targeted drug delivery. The JNP was loaded with folic acid on the polystyrene and doxorubicin on the silica side [76,135]. In another study, gold-mesoporous silica Janus nanoparticles were employed in a bio-imaging system. In these multifunctional JNPs, the nanoparticles were designed to target hepatocellular carcinoma due to the folic acid molecules in their structure. In other words, the folic acid modification of these JNPs makes it possible to act as targeted computed tomography (CT) and imaging agents for diagnosing hepatocellular carcinoma [72]. In another study, López et al. designed asymmetric Janus mesoporous silica particles with two targeted ligands. One is a folic acid molecule for binding to folate receptors of the cell membrane, and the other is triphenylphosphine for binding to the mitochondria membrane. The folic acid ligands facilitate the guided transportation of nanocarriers near the mitochondria, a higher nanoparticle concentration inside the tumor cells [117,136]. This asymmetric designing strategy facilitates the targeted delivery of NPs from cell to organelle compared to conventional symmetric NPs.

Furthermore, in a study by Yang et al., the asymmetric folic acid-coated chitosan NPs were employed as polymeric NPs in the oral cancer imaging system. The fabricated NPs have an excellent capacity for drug loading and an enhanced drug release in cellular lysosomes. In the NP structures, the folic acid ligands facilitate the endocytosis of NPs by attaching to folate receptors on the oral cancer cells [137,138].

## 6. Future Perspective

In this review, various bioinspired nanomaterials for cancer treatments were discussed. Despite the various experimental studies performed on these nanomaterials, only a few clinical studies were performed; therefore, further clinical investigations are needed in order to confirm the advantages and efficacy of these materials in the clinic. Furthermore, these materials mostly have complicated structures with time-consuming and costly production, inhibiting their commercialized production. Additionally, in clinical treatment, high drug concentration in target cells and efficient drug release are needed; therefore, combination therapies must be introduced to increase the affectivity of DDS. For example, ultrasound-guided drug delivery can be a promising method for combined targeted drug delivery, with the utilization of the thermal and mechanical effects of the ultrasound on the employed nanomaterials.

As discussed in this article, asymmetric nanomaterials are widely used in cancer treatment; however, they are just in the primary stages of development and still need to overcome their shortages. First, there is a need to explore an efficient system for fabricating these asymmetric materials on industrial scales. Additionally, some of the asymmetric nanomaterials are currently fabricated in simple asymmetric structures, such as bowl-shaped structures; thus, it is critical to develop methods for the facile and efficient production of these materials in more complex structures and functions. Similar to bioinspired materials, producing asymmetric NPs is also very expensive. Therefore, it is essential to develop efficient and cost-effective methods for fabricating these asymmetric nanomaterials on industrial scales in order to use them in cancer treatments.

## 7. Conclusions

One of the major problems of conventional cancer therapies is their off-target effects on healthy tissues. Recently, with the development of novel nanomaterials, tumor-targeted therapies have been invented in order to achieve more specific cancer treatment with reduced unfavorable side effects of chemotherapic agents on human cells. In this review, we tend to discuss the application of nanomaterials in cancer treatment, emphasizing asymmetric nanomaterials due to their advantages in drug delivery systems, as they can possess multiple functions due to their different surface physiochemical properties or different components. However, the clinical application of nanomedicines has various disadvantages, such as the reduced ability to cross biological barriers and undesirable off-target effects. In this order, the bioinspired materials are developed to minimize the related side effects due to their excellent biocompatibility and higher accumulation therapies. Therefore, this article discussed the asymmetric bioinspired and biomimetic nanomaterials in cancer treatments.

## Figures and Tables

**Table 1:** The comparison of advantages and disadvantages between symmetric and asymmetric nanostructures.

Symmetric Structures	Asymmetric Structures
Lower effective surface area	Multiple functions
Fewer active sites	More active sites
Single function	Larger effective surface area
Free energy effect limits symmetric structures	Stronger synergistic effect
	Distinct properties
	Lower free energy
	More complex assemblies
	Increased number of unsaturated coordination centers
	More mechanic resistance
	Permeability

## Data Availability

All data generated or analyzed during this study are included in this published article.

## Abbreviations

5 FU: 5 fluorouracil; BC: Bacterial Cellulose; BPPE: black pomegranate peel extract; CCMNPs: chitosan-coated magnetic nanoparticles; CD: Cyclodextrins; CDDP: cis diamminedichloroplatinum; CNPs: covalent channel-type nanoparticles; CT; computerized tomography; DDSs: drug delivery systems; DOX: doxorubicin; EGFR: growth factor receptor; FA: folic acid; FGB: Folic acid-gold-bilirubin; JNPs: Janus nanoparticles; LA: lactobionic acid; M-γ-CD: mannose-modified γ-cyclodextrin; MRI: magnetic resonance imaging; MNPs: Magnetic nanoparticles; NIR: near-infrared; NPs: nanoparticles; OSCC: oral squamous cell carcinoma; PAI: photoacoustic imaging processing; PCL: polycaprolactone; PEG: polyethylene glycol; PEI: polyethyleneimine; PGA: poly (glycolic acid); PLA: poly (lactic acid); PLGA: poly (lactic-co-glycolic acid); PMO: periodic mesoporous organosilica; SERS: surface-enhanced Raman scattering; SPR: surface plasmonic resonance; RES: reticuloendothelial system; RG: Regorafenib ROS: reactive oxygen species; TiO2: titanium dioxide; UCNPs: upconverting nanoparticles; VB12: Vitamin B12; VLPs: Virus-Like Particles.
